# 体外循环辅助下难治性胸部肿瘤的外科治疗

**DOI:** 10.3779/j.issn.1009-3419.2018.04.17

**Published:** 2018-04-20

**Authors:** 蓉英 朱, 善州 段, 文涛 杨, 立 施, 福全 张, 勇兵 陈

**Affiliations:** 215004 苏州，苏州大学附属第二医院胸心外科 Department of Cardiothoracic Surgery, the Second Affiliated Hospital of Soochow University, Suzhou 215004, China

**Keywords:** 胸部肿瘤, 体外循环, 外科治疗, Thoracic tumor, Cardiopulmonary bypass, Surgical treatment

## Abstract

**背景与目的:**

回顾性总结体外循环辅助下累及心脏或大血管的难治性胸部肿瘤外科治疗体会。

**方法:**

总结我院2008年1月-2017年5月实施的体外循环下外科手术切除累及心脏或大血管的11例胸部肿瘤患者，分析所有患者的一般情况、临床特征、治疗方法、术后住院时间、并发症及随访结果。

**结果:**

11例患者均在体外循环辅助下手术。肿瘤全部切除者8例，大部分切除者3例，其中1例先行左房内转移性肺平滑肌肉瘤切除，再行右肺中下叶切除术；1例经胸骨正中切口行左肺中央型肺癌切除术；2例同时行肺动脉修补术，3例同时行部分心包切除术，3例同时行肺楔形切除术。所有患者术后症状均得到有效缓解。无院内及术后30天死亡率。3例术后出现肺部感染，于抗生素治疗后恢复。1例淋巴瘤术后6个月复发，1年后死亡；1例心包内纤维肉瘤患者于术后13个月局部复发伴广泛转移，15个月死亡，1例肺平滑肌肉瘤患者于术后15个月发现局部复发灶，予以化疗后缓解。其余8例患者均存活，且计算机断层扫描（computed tomography, CT）检查均未发现明显复发及远处转移。

**结论:**

对于累及心脏或大血管的难治性胸部肿瘤可以实施体外循环辅助下的外科手术治疗，可以提高难治性胸部肿瘤的手术切除率，有效缓解对呼吸、循环功能的影响，明显延长此类患者的生存期。

## Abstract (en)

**Background and objective:**

A retrospective review of the surgical treatment of refractory chest tumors involving the heart or large vessels with cardiopulmonary bypass (CPB).

**Methods:**

To summarize 11 cases of chest tumor patients who had undergone cardiopulmonary bypass surgery from January 2008 to May 2017 in our hospital, and analyze the general condition, clinical characteristics, treatment methods, postoperative hospitalization time, complications and follow-up results of all patients.

**Results:**

All 11 patients were operated with cardiopulmonary bypass. Total resection of tumors in 8 cases and most of the excision in 3 cases. 1 case of left atrial metastatic leiomyosarcoma were excised in the left atrium, and then the right lung resection was performed. 1 case of left lung central lung cancer resection through the median sternum incision. 2 cases underwent pulmonary artery repair at the same time, 3 cases underwent partial pericardiectomy and 3 cases underwent pulmonary wedge resection at the same time. All the patients were effectively relieved after the operation. No death rate in hospital and 30 days after operation. 3 cases of postoperative pulmonary infection were recovered after the treatment of antibiotics. 1 case of lymphoma relapsed 6 months after surgery and died one year later. 1 case of pericardial fibrosarcoma had local recurrence and extensive metastasis at 13 months after operation, and died after 15 months. 1 case of pulmonary leiomyosarcoma were found to have local recurrence 15 months after the operation and were relieved after chemotherapy. The remaining 8 patients survived, and no obvious recurrence and distant metastasis were found in the computed tomography (CT) examination.

**Conclusion:**

The CPB assisted surgical treatment can be performed for patient of refractory chest tumors involving the heart or large vessels. It can improve the surgical resection rate of refractory chest tumors, effectively alleviate the effects on respiratory and circulatory functions, and significantly prolong the survival period of these patients.

对于侵犯到心脏或周围大血管的胸部肿瘤，人们往往惧于其术后的并发症及死亡率而放弃手术。但近年来借助体外循环技术，一些复杂的胸部肿瘤可以得到根治性的切除，从而改善了这类患者的生存质量，甚至延长了生存期。本文总结我院2008年1月-2017年5月收治的体外循环下切除难治性胸部肿瘤11例，总结分析如下。

## 1 临床资料

本文回顾性总结2008年1月-2017年5月在我院实施的体外循环下外科手术切除累及心脏或大血管的11例胸部肿瘤患者资料，包括所有患者的一般情况、临床特征、治疗方法、术后住院时间、术后并发症（术后2天内凝血功能、血常规、血气分析、肾功能指标监测）及术后至今的随访结果。

本组患者中，男性7例，女性4例；年龄30岁-61岁（中位年龄52岁），病史1个月-10个月，临床症状：2例为刺激性咳嗽，其余9例为不同程度的胸闷气促，均无明显肌无力症状。查体：4例有患侧呼吸音减低，其中2例伴患侧哮鸣音，其余无明显阳性体征。所有患者术前均经过详细的病史询问、体格检查、实验室检查、肝胆胰脾肾多普勒彩超检查、心脏彩超检查、肺功能测定及胸部增强三维计算机断层扫描（computed tomography, CT）检查。该组患者根据胸部增强三维CT及心脏彩超表现均有不同程度的侵犯纵隔大血管或心脏，根据影像学表现考虑左侧中央型肺癌1例，右肺下叶恶性肿瘤侵及左房1例，前上纵隔占位5例，左上纵隔占位2例，左下纵隔占位1例，中纵隔占位2例。其中1例入院后第2日突发休克，予气管插管呼吸机辅助通气，血管活性药物持续泵入维持，后因肺部感染，呼吸道管理不佳予以气管切开呼吸机辅助通气，肺部感染得以控制、生命体征基本稳定后行体外循环下胸内肿瘤切除术，其余10例患者术前相关检查无远处转移依据，无绝对手术禁忌症。

手术方法：均采用经胸骨正中切口进胸体外循环下进行，肝素化（3 mg/kg）后，根据肿瘤位置、与纵隔血管侵犯粘连情况选取主动脉或股动脉插管，上下腔静脉或腔房管插管建立体外循环心肺转流（cardiopulmonary bypass, CPB），过程中采用亚低温控制（32 ℃-35 ℃）。使用索林C5型人工心肺机，进口膜肺（Medos公司，德国），管路内预充肝素1 mg/kg，流量50 mL-80 mL/（kg•min），以ACTII型测定仪（Medtronic公司，美国）监测ACT，转流中维持ACT > 480 s。所有患者均经胸骨正中切口，暴露肿瘤，分离肿瘤与心脏、大血管及气管间粘连，完全或部分切除肿瘤。11例患者中肿瘤全部切除者8例，大部分切除者3例，其中1例切开左房摘除左房内转移性肺平滑肌肉瘤，再行右肺中下叶切除术；2例同时行肺动脉修补术，3例同时行部分心包切除术，4例同时行肺楔形切除术。所有患者尽可能保证肿瘤切除彻底，包括切除全部肿瘤组织、肿瘤周围脂肪组织、累及的纵隔胸膜、肺、心包等。停机后按1.0-1.5:1鱼精蛋白中和体内肝素。复查及随访：嘱患者术后1个月复查胸部X线片；无异常者术后每3个月-6个月复查一次胸部X线片或胸部CT；术后2年后改为每年复查一次胸部CT。3例异地患者主要以电话随访、当地医院检查的方式进行随访。

## 2 结果

11例患者中左肺鳞癌累及肺动脉主干1例，右下肺平滑肌肉瘤累及左心房1例，引起左房堵塞致循环不稳定（图 1-图 3），心包腔内纤维肉瘤1例，侵袭性纤维瘤1例（图 4）；胸腺瘤4例，呈侵袭性生长，其中2例累及心包及主肺动脉，1例累及升主动脉及无名静脉，胸腺癌2例，累及主肺动脉；淋巴瘤1例。所有患者手术顺利，平均CPB时间99.4 min（65 min-154 min），术后平均重症加强护理病房（intensive care unit, ICU）监护时间4.3 d（1 d-10 d），术后平均住院时间22 d（14 d-29 d）。所有患者术后症状均得到有效缓解。无院内及术后30天死亡率。3例术后出现肺部感染，于抗生素治疗后恢复，均未出现需要再次气管插管或气管切开的呼吸功能不全。所有患者术后早期均有凝血指标延长，但术后1天均恢复，未出现需要二次开胸止血的病例。9例患者术后肾功能指标监测正常。术后2例大部分切除患者及侵袭性纤维瘤患者行瘤区放疗。术后随访5个月-94个月，1例心包内纤维肉瘤患者于术后13个月局部复发伴广泛转移，15个月死亡，1例肺平滑肌肉瘤患者于术后15个月发现局部孤立病灶，按GP方案（吉西他滨+顺铂方案）同步放化疗2周期后复查胸部CT提示病灶吸收。其余8例患者均存活，且CT检查均未发现明显复发及远处转移。

**1:**
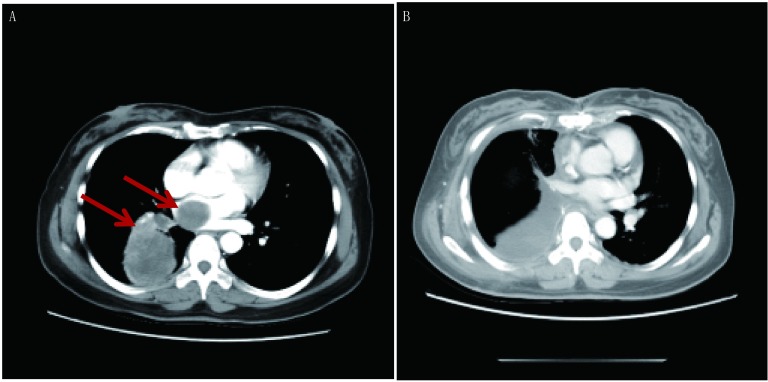
右肺平滑肌肉瘤累及左心房（箭头示），术前及术后早期对比（肿瘤全部切除）。A：术前；B：术后3周。 Right lung leiomy-osarcoma involving the left atrium (Arrow), preoperative and postoperative CT scan (The tumors were removed completely). A: preoperative; B: 3 weeks after operation. CT: computed tomography.

**2:**
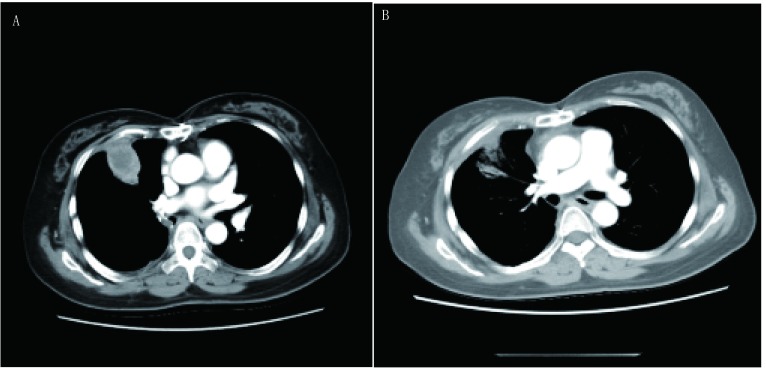
右肺平滑肌肉瘤累及左心房术后CT复查。A：术后15个月，肿瘤复发；B：化疗2个月后（术后17个月），肿瘤明显缩小。 Postoperative CT scan of the right lung leiomyosarcoma involving the left atrium. A:Tumor recurrence 15 months after operation; B:After 2 months of chemotherapy (17 months after the operation), the tumor was significantly reduced.

**3:**
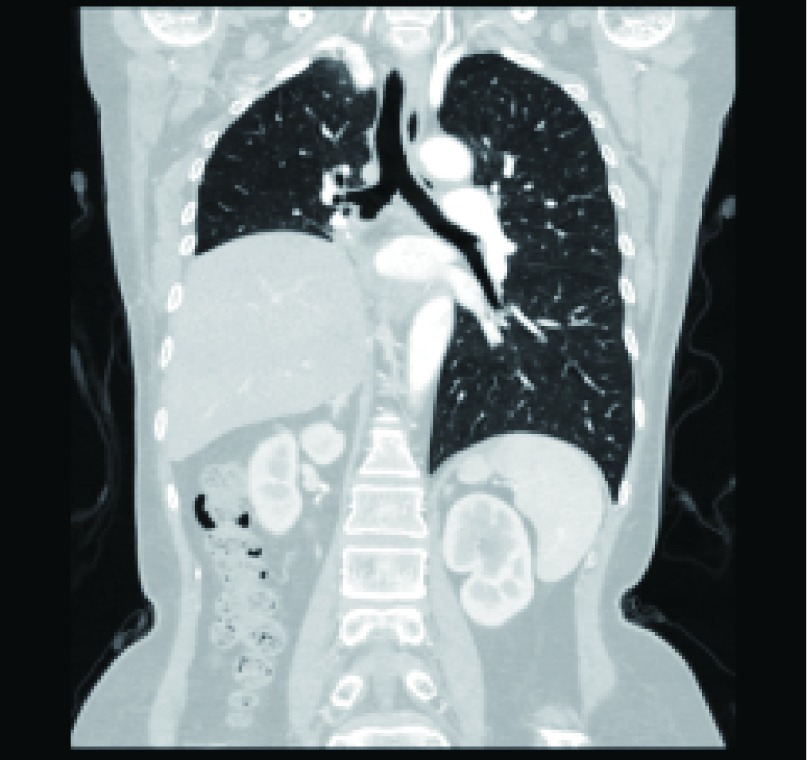
术后8年（右侧膈肌抬高，无肿瘤复发） 8 years after operation (Right diaphragm raising and no tumor recurrence)

**4:**
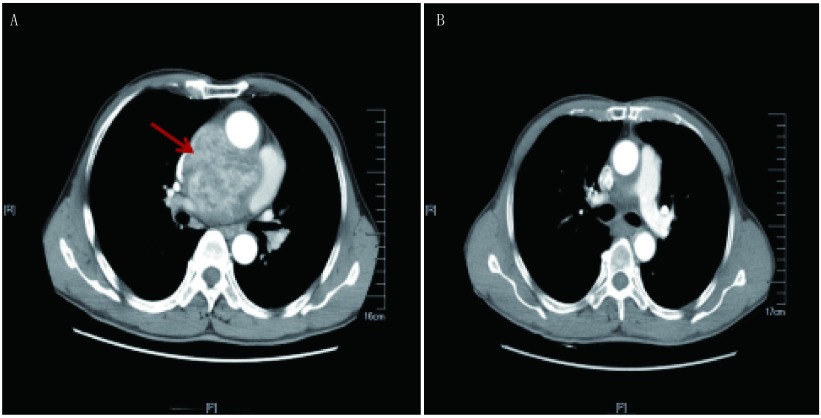
侵袭性纤维瘤术前及术后CT检查。A：侵袭性纤维瘤术前CT检查（箭头示)；B：术后21个月复查CT。 Preoperative and postoperative CT scan of invasive fibroma. A:Preoperative CT scan of invasive fibroma (arrow); B:CT scan of 21 months after operation.

## 3 讨论

胸部肿瘤最主要治疗方法仍是手术，特别是根治性手术切除可以显著改善除恶性肿瘤和无远处转移的患者的预后^[1-4]^。但当肿瘤局部生长侵犯、包绕周围血管、心脏等纵隔内重要器官时，就大大增加了手术切除难度。体外循环技术在手术切除累及心脏、大血管等胸部肿瘤中具有独道的优势，但由于术后易导致凝血功能障碍、肾功能损害等并发症^[5]^，目前仅有少数相关病例报道。本组11例患者均累及心脏、大血管，采用体外循环技术，完整切除肿瘤8例，大部分切除3例，经过积极处理均顺利恢复。

积极的术前评估和术前准备可以显著降低术后并发症发生率^[6]^。术前全面评价患者身体状况，包括心肺功能、肝肾功能、凝血功能，了解手术的耐受性，排除严重的肝肾功能不全及其他不耐受手术的疾病，治疗并控制慢性病如：糖尿病、高血压等。在本组病例中，胸部增强CT及三维重建在术前全面评估肿瘤, 明确肿瘤性质、形态、边界、瘤内情况和周围血管结构位置情况起了不可忽视的作用，其中1例患者经连续增强薄层CT扫描考虑系右肺肿瘤经肺静脉形成瘤栓伸向左房，故在手术时借助体外循环保证了术中完整切除肿瘤及转移瘤^[7]^（图 1），该患者在术后近8年后的胸部CT随访中未见肿瘤复发（图 3）。磁共振则在软组织及血管显影上更占优势，特别是对明确肿瘤侵犯心肌、大血管及胸壁情况上，另外对于碘剂过敏的患者也可选择磁共振行术前评估^[6]^。术前我们常规完善心脏彩超评估心脏功能及纵隔肿瘤压迫累及心脏情况，对于明确肿瘤侵犯至心腔内的，则进一步行经食道心脏超声评估。术前手术医师、麻醉医师及体外循环师等多学科讨论制订相关手术方案，更利于手术进行。有研究^[8]^认为非计划内的急诊体外循环辅助手术并不能使患者获益。

手术切口选择本身不能改变患者预后，但适宜的手术切口可以充分暴露肿瘤以利于完全切除肿瘤^[6]^。在本组病例中均采用了经胸骨正中切口进胸，对于前纵隔肿瘤暴露清楚，且方便快速建立体外循环通路。巨大的前纵隔肿瘤在诱导麻醉时可能因压迫气管及大血管引起心跳呼吸骤停，此类高风险患者可在术前评估后宜选用局麻下先行股-股转流。本组病例胸部肿瘤均累及心脏或大血管，瘤体较大，且与主动脉、肺动脉关系紧密甚至呈浸润表现（图 1、图 4），分离较困难，易出现大出血，采用CPB以后，心脏得以适当放空，主、肺动脉张力减低，有利于肿瘤分离，同时，心肺转流时亚低温保证了各远端脏器的血流灌注，消除了术者为暴露肿瘤所致心脏大血管受压带来的低血压、低灌注方面的担忧，方便了肿瘤的暴露，提高了肿瘤的切除几率。本组病例中1例系心包内肉瘤与心脏粘连致密及1例术前已行2次新辅助化疗，术中见瘤体边界与周围组织粘连致密，还有一例胸腺癌未能全部切除外，余8例患者均全部切除（图 1、图 4）。巨大的胸内肿瘤，因其局部粘连广泛，肿瘤血液供应丰富，分离肿瘤时创面渗血多，机体失血量大，往往需输注大量血液成分，而心肺转流的施行可以使术野血液重吸收利用，减少失血及库存血输注，降低围术期相关并发症。

Ried等^[9]^认为使用体外循环技术完整的肿瘤切除往往伴随着血胸、重症肺炎致呼吸功能不全、急性肾功能不全等术后并发症的发生，在本组研究中，仅3例出现肺部感染经积极治疗后恢复，未出现需要再行气管插管或气管切开的呼吸功能不全，预防性使用抗生素可以减少术后肺部感染发生。术后24 h内11例患者均有不同程度凝血功能指标延长但经鱼精蛋白滴定中和肝素，未出现需要二次开胸止血的情况，选用合适的氧合器、体外循环管道、术中亚低温控制、优化的预充方案选择可以明显减少对机体凝血功能损伤^[10]^。严格控制体外循环时间，保持肾脏灌注，可以显著降低术后急性肾功能不全的发生。而心肺转流过程中血液回收是否增加肿瘤细胞血行播散，目前尚无充分证据，有研究^[11, 12]^表明心肺转流中常规使用的肝素、抑肽酶对肿瘤的浸润转移能起抑制作用。在本组研究中8例患者均无肿瘤复发和转移，仅行肿瘤部分切除的1例患者在术后15个月出现肿瘤复发转移，1例出现局部复发，于同步放化疗后瘤体消失。在Turbendian等^[2]^的研究队列中，使用CPB辅助手术的肉瘤患者中局部复发率等同远处转移率，是否提示CPB并未增加肿瘤血行播散，有待于大样本研究证实。

综上所述，尽管累及心脏大血管的难治性胸部肿瘤手术风险高，但经过术前完善检查及准备，多学科讨论制定周密手术方案，使用CPB辅助手术可以显著提高难治性胸部肿瘤的手术切除率，有效缓解对呼吸、循环功能的影响，提高生活质量，甚至延长此类患者的生存期。
